# Safe temperature range for intraoperative and early postoperative continuous hyperthermic intraperitoneal perfusion in a swine model of experimental distal gastrectomy with Billroth II reconstruction

**DOI:** 10.1186/1479-5876-11-181

**Published:** 2013-07-29

**Authors:** Sheng Li, Yong-lei Zhang, Jia-yuan Sun, Ya-wei Hua, Pei-hong Wu

**Affiliations:** 1 State Key Laboratory of Oncology in South China, 510060, Guangzhou, Guangdong, P.R China; 2 Department of Medical Imaging & Interventional Radiology, Sun Yat-sen University Cancer Center, 510060, Guangzhou, Guangdong, P.R China; 3 Henan Province Cancer Hospital, Guangzhou, P.R China; 4 Department of Radiotherapy, Sun Yat-sen University Cancer Center, 510060, Guangzhou, Guangdong, P.R China

**Keywords:** Temperature, Safety, Swine model, Continuous hyperthermic intraperitoneal perfusion, Anastomotic healing, Abdominal adhesion, Intraoperative, Early postoperative

## Abstract

**Background:**

The current study sought to investigate the safety of intraoperative and early postoperative continuous hyperthermic intraperitoneal perfusion (IEPCHIP) at different temperatures in a swine model of experimental distal gastrectomy with Billroth II reconstruction.

**Methods:**

Thirty pigs were randomly divided into 5 groups. Two groups were used as the control groups (groups A1 and A2), and 3 groups were used as the perfusion groups (groups B, C and D). Pigs in group A1 received distal gastrectomy with Billroth II reconstruction only. Pigs in groups A2, B, C and D received the same surgery as group A1, followed by IEPCHIP at 37 ± 0.5°C, 42.5 ± 0.5°C, 43.5 ± 0.5°C or 44.5 ± 0.5°C, respectively. The perfusion time was assessed for each pig in group A2 as well as in the perfusion groups, and the perfusions were performed twice for each group. The first perfusion was conducted intraoperatively, and the second perfusion was initiated 1 day after surgery. Data concerning vital signs and hepatic and renal function were collected. Parameters concerning anastomotic healing, the pathology of the anastomotic tissue and abdominal adhesion were compared.

**Results:**

The vital signs and hepatic and renal functions of the pigs in groups A1, A2, B and C were not significantly affected by this procedure. In contrast, the vital signs and hepatic and renal functions of the pigs in group D were significantly affected. Compared to the pigs in groups A1, A2 or B, the anastomotic bursting pressure, breaking strength and hydroxyproline content in group C and D pigs were significantly lower. No significant differences were observed in these parameters between groups A1, A2 and B. Abdominal adhesion was more severe in group D pigs. Collagen deposition in group A1, A2 and B pigs was dense in the anastomosis, and inflammatory cell infiltration was observed in group D.

**Conclusions:**

IEPCHIP at 42.5 ± 0.5°C was safe and caused minimal impairments. However, anastomotic healing was affected by perfusion at 43.5 ± 0.5°C and 44.5 ± 0.5°C, and abdominal adhesion was most severe in the group D animals, which were perfused at 44.5 ± 0.5°C.

## Background

More than 900,000 new cases of gastric cancer are diagnosed worldwide each year, and 42% of these cases occur in China [1]. In addition, more than 30% of advanced gastric cancers coexist with peritoneal dissemination, and the median survival time for these patients is approximately 6 months [2,3].

Even after radical gastrectomy, more than 50% of patients with advanced gastric cancer may die of peritoneal recurrence [3,4]. In recent years, the improved survival associated with hyperthermic intraperitoneal perfusion chemotherapy for peritoneal carcinomatosis of gastric cancer [5,6] has been verified by both experimental [7] and clinical research [5,8]. Moreover, cytoreductive surgery plus hyperthermic intraperitoneal perfusion chemotherapy has become the priority treatment for gastric cancers with peritoneal carcinomatosis [9], and survival can be prolonged by intraoperative and early postoperative intraperitoneal chemotherapy [10,11]. This treatment may result in the timely elimination of free cancer cells resulting from operative factors or spontaneous dissemination. Thus, hyperthermic intraperitoneal perfusion is considered effective for preventing and/or treating peritoneal carcinomatosis and promoting survival [5,12].

However, hyperthermic intraperitoneal perfusion chemotherapy increases perioperative complications and mortality in cases of advanced gastric cancer [13-18]. The morbidity and mortality rates from 1999 to 2010 were between 12% and 66.3% and between 0% and 12.5%, respectively [3].

Early perfusion is thought to lead to an added increase in the risk of postoperative complications and mortality. Accordingly, the safety of early hyperthermic perfusion has become an issue of importance. Anastomotic repair, following reconstruction of the digestive tract, is considered one of the most crucial factors involved in postoperative safety. When anastomotic malunion leads to leakage, it may trigger abdominal infection, adhesive bowel obstructions, anastomotic stricture and even septic shock [14-16]. Although reduced life quality should not be a reason to deny this therapy [19], some investigators believe that hyperthermia plus chemotherapeutics affect wound healing [20]. Furthermore, hyperthermic intraperitoneal perfusion chemotherapy after gastrointestinal surgery does increase the likelihood of anastomotic leakage and perioperative mortality [17,21].

Generally, cancer cells are effectively eliminated by hyperthermic perfusion if the temperature exceeds 42°C [22,23], although hyperthermia greater than 45°C leads to irreversible hepatocyte damage [24]. Klaver and coworkers examined adjuvant treatment after cytoreductive surgery for peritoneal carcinomatosis in a rat model, and their investigations of hyperthermic intraperitoneal perfusion at 37 or 41°C require further study [25].

Moreover, few studies have been performed to examine the safe and optimal temperature range for intraoperative and early postoperative continuous hyperthermic intraperitoneal perfusion (IEPCHIP) in the treatment of peritoneal carcinomatosis of gastric cancer. Thus, we conducted an investigation of IEPCHIP in a swine model of experimental distal gastrectomy with Billroth II reconstruction to examine the safety of this procedure at a temperature range of 37 to 45°C.

## Methods

This study was performed according to the principles of the Declaration of Helsinki, and the institutional review boards of the respective institutions reviewed and approved its design.

### Model building and management

Thirty healthy and mature Bama Minipigs were used (15 females and 15 males). The animals were provided by the mini pig incubator from Taizhou City, Jiangsu Province. The average weight of these animals was 20 ± 2 kg.

All pigs were fasted for 12 h prior to surgery. The procedure was performed under intravenous and inhaled anesthesia. The same team of surgeons performed all of the procedures to ensure technical uniformity.

**Figure 1:**
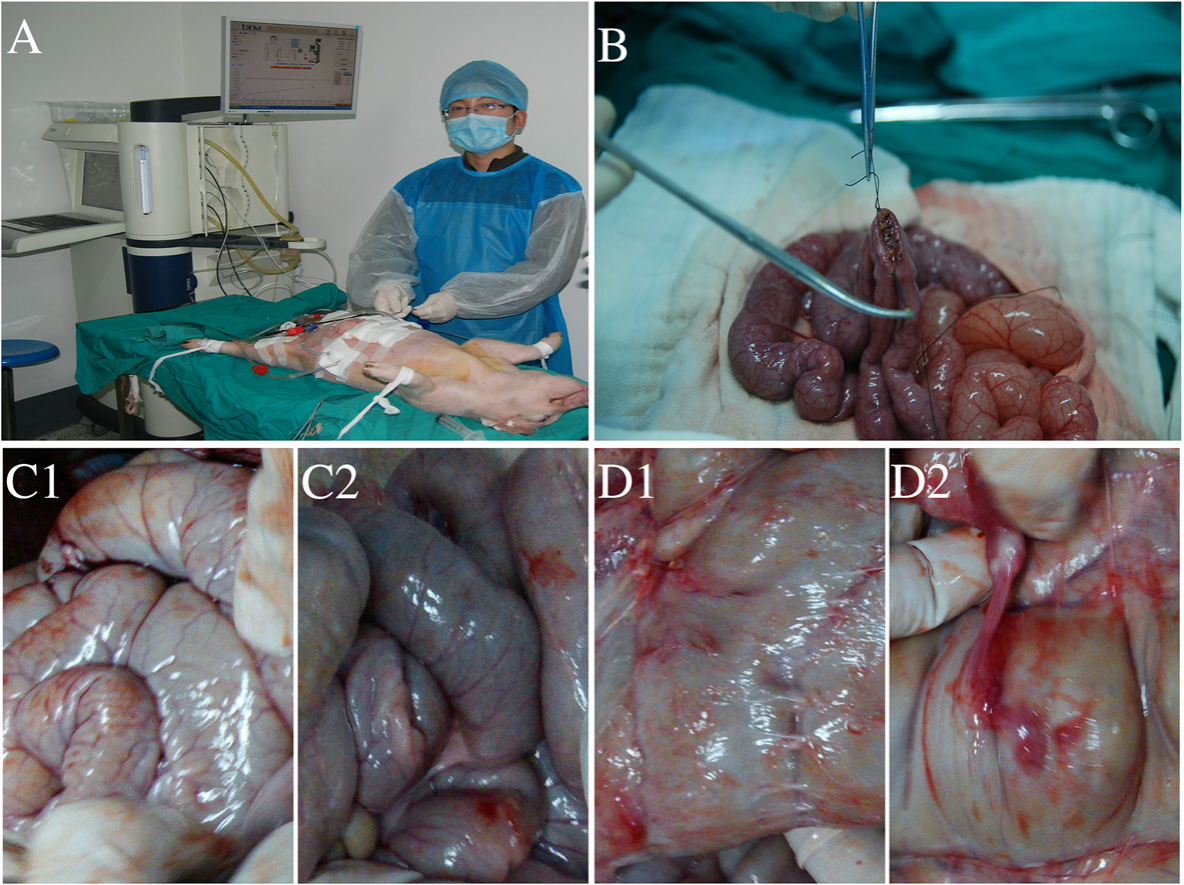
**The procedure and postoperative adhesion. A**, Enteroenterostomy. **B**, Hyperthermic perfusion in the swine model. **C1** and **C2**, No postoperative adhesion was observed in groups **A**2 and **B**. **D1** and **D2**, Postoperative filmy and dense adhesion was observed in group **D**.

The Bama pigs were randomly divided into 5 groups. Groups A1 and A2 served as the control groups, and groups B, C and D represented the hyperthermic perfusion groups. Pigs in group A1 underwent distal gastrectomy with Billroth II reconstruction (Figure 1A). The other groups received IEPCHIP after undergoing the same procedure as group A1.

Preoperatively, the abdomen was prepared by shaving. After satisfactory anesthesia was achieved under sterile conditions, we performed a distal stomach resection and constructed a gastrojejunostomy and an end-to-end jejunojejunostomy through a median incision using 3–0 silk sutures (Ethicon, Norderstedt, Germany). In groups A2, B, C and D, two outflow catheters were placed beneath the diaphragm, and 2 inflow catheters were placed in the pelvic cavity of each pig (Figure 1B). The catheters were fixed to surrounding skin with 1–0 silk sutures (Ethicon, Norderstedt, Germany). All skin incisions were then sutured to close the abdomen.

### The IEPCHIP procedure

The animals in groups A2, B, C and D were connected to the hyperthermic intraperitoneal perfusion treatment system (BR - TRG Type 1, Guangzhou Poris Medical Technology Co., LTD, Guangzhou, China). The intraperitoneal perfusion circuit was established with a constant temperature, and dynamic temperature monitoring was performed near the inflow and outflow catheters, the anus and the mouth using electronic thermometers. Two temperature probes were added into the in- and outflow catheters in the abdominal cavity and were attached to the heat exchanger to control the thermal distribution. Through the path where the inflow and outflow catheters enter the abdomen, all perfusion solutions were monitored in real time by inserting a thermometer into the abdomen to measure the temperature of the perfusate. The perfusate of groups A2, B, C and D were preheated to 37°C, 42.5°C, 43.5°C and 44.5°C, and the water temperature difference between the inflow and outflow catheters was controlled to a level ≤ 0.5°C. Thus, the perfusion temperature in the peritoneal cavity was maintained at 37 ± 0.5°C, 42.5 ± 0.5°C, 43.5 ± 0.5°C and 44.5 ± 0.5°C for groups A2, B, C and D, respectively. The perfusion parameters included a perfusate volume of 2,500 ml saline and a flow rate 1.5 l/min. Each pig in group A2 and the perfusion groups received 60 min of perfusion, and the perfusions were performed twice. The starting time for the first perfusion was when the surgery was finished and the abdomen had been closed. The second perfusion was initiated 1 day after the surgery. We gently massaged the abdominal wall during perfusion to equalize the distribution of the perfusion fluid.

After the perfusion, we used multiple bellybands to provide protection to the abdominal incision. No pigs received food for 24 h postoperatively, and all animals were intravenously treated with 80,000 IU gentamicin and 0.5 g metronidazole in a glucose and sodium chloride solution at a dose of 40 ml/kg. On the third day, a no-slag liquid diet was administered, and on the sixth day, the animals were given a semi-fluid diet and were gradually transitioned to a normal diet. Postoperative intraperitoneal hyperthermic perfusion was also performed in pigs under systemic anesthesia with their legs fastened. Tramadol at 30 mg per day was administered to relieve pain; this treatment was continued for 3 successive days after surgery.

### Observed parameters

We measured respiratory frequency, blood pressure, anal temperature and heart rate in the pigs 2 hours before, during and 2 hours after the procedure. We also observed the postoperative condition of the experimental animals, including defecation, diet, activity and weight changes, and we classified the performance of each animal after surgery.

Peripheral venous blood was extracted from all of the animals prior to surgery and 1 day, 3 days, 7 days and 14 days after the operation. Glutamic-pyruvic transaminase (ALT), albumin (ALB), total bilirubin (TBIL), blood urea nitrogen (BUN) and creatinine (CR) levels were evaluated.

The animals were sacrificed 14 days after the procedure, at which time an autopsy was performed.

### Measurement of anastomotic bursting pressure, breaking strength and hydroxyproline content

After the anastomotic segments were cleared with saline, the anastomotic bursting pressure and breaking strength were measured. After cutting both ends of the anastomosis, two catheters were inserted 3 cm from either side of the anastomosis; then, two ligations were made 3 cm proximal and distal to the anastomosis by using 1–0 silk sutures. The catheters were connected to an infusion pump and a sphygmomanometer with the sleeve belt removed. Steady infusion with air allowed for a uniform increase in the pressure at the anastigmatic site. The pressure of mercury at the anastomotic site was increased by approximately 1 mmHg/sec. When the mercury level did not rise or fall, the pressure reading was recorded as the bursting pressure (mmHg), which represents the peak intraluminal pressure just prior to leakage. In addition, the fracture location was recorded. The jejunum bursting pressures of normal adult pigs were used as controls. Next, the ligation to the connection pipe was removed, and a longitudinal incision on the anastomosis was made. One end of the resulting anastomosis strip was fixed, and the other end was attached to a spring. The tension was then increased in 0.098 N/s increments until the anastomosis broke. At the breaking point, the tension was noted, and this tension was defined as the breaking strength (kg).

Hydroxyproline provides an index of collagen concentration, as it is the most common amino acid in collagen. A chemical colorimetric method, with analytically pure reagents, was used to calculate the hydroxyproline content according to the following formula: hydroxyproline content (μg/mg wet weight) = (absorbance of spectrophotometer tube - blank tube absorbance/standard tube absorbance - blank tube absorbance) × standard substance content (5 μg/ml) × hydrolysate overall volume (10 ml) / wet weight of organization (mg).

### Pathological examination

The anastomotic tissue was excised and fixed in a 4% formaldehyde solution by using routine and standard procedures. Serial 5-μm sections of paraffin blocks were prepared and stained with hematoxylin-eosin (HE). These specimens were evaluated with light microscopy.

### Statistical analysis

All experimental data were analyzed with SPSS18.0 Statistical Software (SPSS Inc, Chicago, IL, USA). The data concerning liver and kidney functions were expressed as the means ± SD. Differences among the groups were analyzed and compared using an analysis of variance and the Newman-Keuls test. *P* values less than 0.05 were considered statistically significant.

## Results

No major complications occurred during the surgery or the perfusion procedures.

### Abdominal adhesion and anastomosis

Adhesion was not detected in groups A1, A2 (Figure 1, C1) or B (Figure 1, C2). Only 1 case with membranous adhesion was observed in group C. Membranous adhesion and dense adhesion were observed in 3 pigs in group D (Figure 1D1, D2).

**Figure 2:**
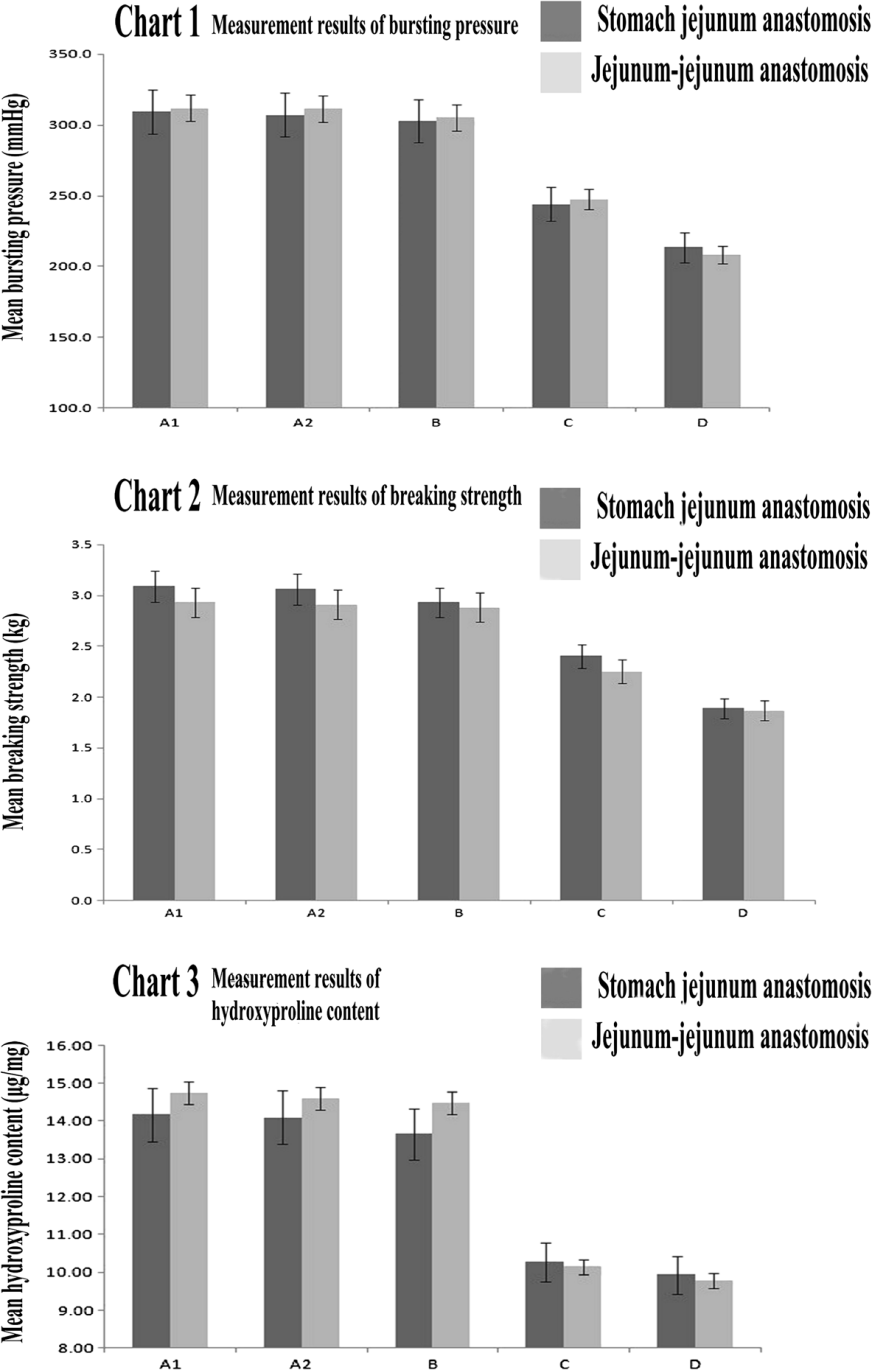
**Three charts depicting measurements of wound healing.** Chart 1, Measurement results for bursting pressure. The bursting pressures of the stomach jejunum anastomosis and the jejunum-jejunum anastomosis were not significantly different between animals in group B and group A (P > 0.05). The bursting pressure of the anastomosis was significantly different for animals in groups C and D (P < 0.05) as compared to groups A1 and A2. Chart 2, Measurement results for breaking strength. The breaking strength of the stomach jejunum anastomosis and the jejunum-jejunum anastomosis was not significantly different between animals in group B and groups A1 and A2 (P > 0.05). The breaking strength of the anastomosis was significantly different for the animals in groups C and D (P < 0.05) as compared to groups A1 and A2. Chart 3, Results of hydroxyproline content measurements. The hydroxyproline content of the stomach jejunum anastomosis and the jejunum anastomosis was not significantly different between animals in group B and groups A1 and A2 (P > 0.05). The hydroxyproline content of the anastomosis was significantly different for animals in groups C and D (P < 0.05) as compared to groups A1 and A2.

The average bursting pressure, breaking strength and hydroxyproline content of the stomach-jejunum anastomosis and jejunum-jejunum anastomosis in groups C and D were less than those values observed in groups A1, A2 and B (Figure 2).

### Pathology

**Figure 3:**
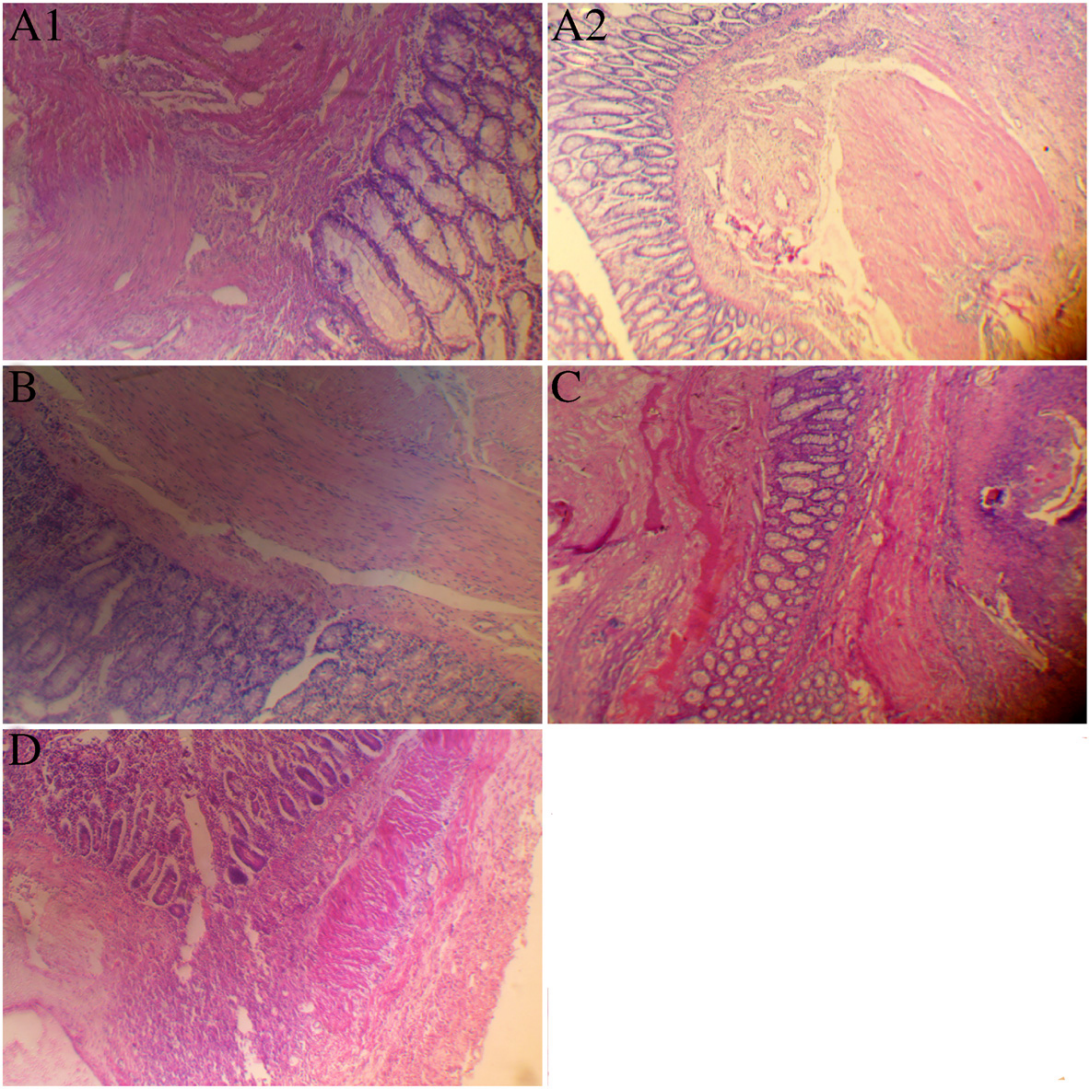
**Pathological examination of anastomotic tissue. A1** and **A2**, Collagen was deposited densely in the anastomosis, with better repair of the mucous layer in groups **A1** and **A2**. **B**, Collagen was deposited densely in the anastomosis of group **B** animals. **C**, Reduced and irregular collagen deposition was observed in the anastomosis of group **C** animals. **D**, Reduced and irregular collagen deposition and inflammatory cells infiltration was observed in the anastomosis of group **D** animals.

Collagen fiber formation inside the anastomosis was visible following HE staining. Compared with that of groups B, C and D, collagen was more densely deposited in the anastomosis of groups A1 and A2, with better granulation and tissue formation, and the repair of the mucous layer was superior in the 2 groups (Figure 3, A1 and A2). In group B, collagen deposition was dense in the anastomosis (Figure 3B), and irregular collagen deposition was present in smaller quantities in group C than in group B (Figure 3C). Collagen deposition was least dense in group D and was accompanied by inflammatory cell infiltration (Figure 3D).

### Perioperative vital signs

Compared to the values obtained prior to perfusion, the body temperature of the pigs in groups A1 and A2 was stable, as were the heart rates, respiratory rates and mean arterial pressures during the procedure. In addition, after perfusion of the animals in groups B and C, the body temperatures increased by an average of approximately 1°C, the heart rate increased to an average of 105 beats per min, the respiratory rate increased to 35 times per min, and the mean arterial pressure decreased by approximately 25 mmHg. Two hours after the procedure, the vital signs reached normal levels in these animals. In group D, the average temperature rose by 1.5°C, the heart rate increased to approximately 120 beats per min, the respiratory rate rose to 40 times per min, and the mean arterial pressure decreased by approximately 35 mmHg. The vital signs of these animals did not return to normal levels within 2 h of the procedure.

### Postoperative recovery

All pigs were prone for most of the first day. All of the pigs in groups A1, A2, B and C were able to stand and walk 1 day after the procedure. After 2 days, the animals were able to drink and eat. After the procedure, only 1 pig in group C vomited twice on the first day. Furthermore, the mental status and performance status of the animals were fair. However, the animals in group D were unable to stand on the day after the procedure, and three pigs groaned continuously. Two days later, the animals could only drink, but on the third day, these pigs began eating. In group D, one pig experienced vomiting 2 times at the first day; and another pig vomited 6 times/day for 3 successive days.

### Liver and renal function

The levels of ALT, TBIL and ALB in the pigs in groups A1 and A2 remained stable. At perfusion temperatures of 37 ± 0.5°C, 42.5 ± 0.5°C and 43.5 ± 0.5°C, a 60-min continuous hyperthermic intraperitoneal perfusion had no significant influence on liver or kidney function. After 1 week, the group A1 and A2 pigs had recovered to normal levels. In contrast, at a temperature of 44.5 ± 0.5°C, a 60-min continuous hyperthermic intraperitoneal perfusion seriously impaired the animals’ liver and kidney functions. This impairment in kidney and liver function lasted for at least 2 weeks. Parameters related to liver and renal functions are shown in Tables 1, 2 and 3 for the pigs in groups B, C and D.

**Table 1:** Changes in the liver and renal function of the pigs in group B

**Before the perfusion**		**After the perfusion**				
		**1 d**	**3 d**	**5 d**	**7 d**	**14 d**
ALT	65.65 ± 5.34	70.65 ± 4.11★	78.65 ± 4.75■	98.45 ± 3.64■	71.48 ± 5.35★	63.29 ± 4.58★
TBIL	8.53 ± 1.01	10.12 ± 2.23★	17.36 ± 3.32■	11.75 ± 3.79★	10.19 ± 3.42★	9.87 ± 1.44★
ALB	30.01 ± 2.47	29.02 ± 1.13★	25.83 ± 1.42■	26.91 ± 2.35★	29.42 ± 2.34★	30.02 ± 1.23★
CR	58.12 ± 5.24	60.72 ± 4.01★	64.49 ± 5.06★	69.92 ± 2.45■	63.76 ± 6.01★	59.12 ± 3.19★
BUN	4.59 ± 1.79	4.98 ± 2.34★	7.29 ± 1.05★	10.02 ± 1.14■	7.09 ± 2.23★	5.03 ± 2.21★

*ALT* Alanine aminotransferase, *TBIL* Serum total bilirubin, *ALB* Albumin, *CR* Creatinine, *BUN* Blood urea nitrogen.

**Table 2:** Changes in the liver and renal function of the pigs in group C

**Before the perfusion**		**After the perfusion**				
		**1 d**	**3 d**	**5 d**	**7 d**	**14 d**
ALT	66.24 ± 5.40	71.32 ± 2.32★	80.11 ± 4.26■	102.31 ± 3.23■	72.35 ± 6.19★	68.71 ± 3.23★
TBIL	8.45 ± 0.94	11.1 ± 03.02★	15.45 ± 2.19■	18.75 ± 1.08■	11.65 ± 4.01★	10.09 ± 2.13★
ALB	29.34 ± 2.23	28.19 ± 3.41★	27.43 ± 2.10★	26.39 ± 4.02★	28.39 ± 3.05★	29.33 ± 1.92★
CR	57.73 ± 5.48	61.45 ± 3.76★	65.34 ± 6.51★	70.24 ± 4.19■	64.95 ± 5.59★	60.23 ± 4.76★
BUN	4.58 ± 1.83	5.02 ± 1.45★	7.31 ± 2.97★	9.65 ± 1.75■	8.63 ± 2.12■	5.12 ± 1.39★

*ALT* Alanine aminotransferase, *TBIL* Serum total bilirubin, *ALB* Albumin, *CR* Creatinine, *BUN* Blood urea nitrogen.

**Table 3:** Changes in the liver and renal function of the pigs in group D

**Before the perfusion**		**After the perfusion**				
		**1 d**	**3 d**	**5 d**	**7 d**	**14 d**
ALT	65.98 ± 4.90	70.32 ± 3.90★	76.45 ± 5.18■	89.65 ± 4.23■	95.27 ± 6.03■	84.72 ± 5.03■
TBIL	8.39 ± 0.87	12.03 ± 0.87■	22.42 ± 1.12■	20.63 ± 1.43■	14.23 ± 2.01■	10.12 ± 1.90★
ALB	30.02 ± 2.14	29.12 ± 1.90★	28.07 ± 1.23★	27.34 ± 2.17★	25.54 ± 1.47■	24.35 ± 1.59■
CR	57.43 ± 5.28	68.23 ± 5.12■	79.24 ± 6.23■	88.47 ± 4.09■	80.92 ± 5.35■	70.22 ± 4.01■
BUN	4.66 ± 1.79	8.03 ± 1.32■	9.23 ± 2.03■	10.12 ± 1.92■	9.04 ± 1.97■	8.64 ± 2.24■

Notes: in comparison to the values obtained prior to perfusion, ★ = no significant difference(P > 0.05) and ■ = significant difference (P < 0.05).

*ALT* Alanine aminotransferase, *TBIL* Serum total bilirubin, *ALB* Albumin, *CR* Creatinine, *BUN* Blood urea nitrogen.

## Discussion

Intraoperative hyperthermic intraperitoneal chemotherapy (IHIC) alone and IHIC combined with early postoperative intraperitoneal chemotherapy have been performed in previous studies [10,26-28]. Improved survival has been reported in these studies, albeit with certain levels of mortality and severe complications [11,29]. The goal of IEPCHIP is to kill free cancer cells and remove micro lesions at an early time after cytoreductive surgery.

During the perfusion process, the vital signs of the animals studied varied across the perfusion groups. The pigs in groups A1, A2, B and C recovered quickly and ate and drank sooner after surgery, than the pigs in group D. As demonstrated above, the 44.5 ± 0.5°C perfusate had a significant and lasting impact on the animals’ vital signs and diet.

The liver can also be affected by anesthesia and hyperthermic intraperitoneal chemotherapy. In one previous study, the hepatic and renal functions of mice in a hyperthermic intraperitoneal chemotherapy group treated at 41°C were not significantly different from a normal temperature group receiving an application of a paclitaxel and beta-cyclodextrin formulation [30]. In these studies, heat stress was thought to cause the liver damage, which was aggravated over time and was thought to arise from a series of complex oxidative stress responses [31]. In the present experiment, the changes in hepatic and renal function in groups A2, B and C were reversible. Perfusion at 37 ± 0.5°C, 42.5 ± 0.5 and 43.5 ± 0.5°C also caused related physiological parameters to rise transiently and mildly. Nevertheless, perfusion at 44.5 ± 0.5°C cased these parameters to change substantially, resulting in apparent hepatic and renal dysfunction. The 2 perfusions performed at 44.5 ± 0.5°C induced a lasting and obvious impairment to liver and renal functions.

After hyperthermic intraperitoneal chemotherapy, 4% of a group of patients with gynecological cancer developed intestinal obstruction due to intestinal adhesion [32], and the majority of these obstructions were related to intra-abdominal tumor progression. Intestinal obstruction may be even more common after surgery and hyperthermic intraperitoneal chemotherapy for gastrointestinal cancer. In the current study, abdominal adhesion was not observed in groups A1, A2 and B and was considered mild in group C. However, perfusion at 44.5 ± 0.5°C induced severe adhesion. This level of hyperthermia likely damaged the gastrointestinal serous and parietal peritoneum, which led to the secretion of inflammatory substances and fibrous proteins and the formation of abdominal adhesion. A low average anastomotic bursting pressure and breaking strength has been shown to be indicative of poor anastomosis healing [20,33]. Aarts et al. [34] found that in comparison to cytoreductive surgery alone, mice receiving cytoreductive surgery plus hyperthermic intraperitoneal chemotherapy appeared to have a significant decline in the average anastomotic bursting pressure of the gastrointestinal tract. However, in the present study, there was no intestinal obstruction or anastomotic leakage observed in any of the pigs.

Although no obvious anastomotic leakage occurred, the anastomotic bursting pressure and breaking strength declined gradually with the rise of the perfusion temperature. Particularly, when the perfusion temperature was 43.5 ± 0.5°C or higher, these 2 parameters were significantly lower than those observed in groups A1 and A2. Compared to groups A1 and A2, the hydroxyproline content in the stomas of the perfusion groups decreased, and this effect was significant when compared to the effects of 43.5 ± 0.5°C perfusion. Nevertheless, neither the average anastomotic bursting pressure nor the hydroxyproline content of group B was lower than that observed in the control groups. This result suggests that perfusion at 42.5 ± 0.5°C does not increase the risk of anastomotic leakage. As demonstrated above, hyperthermal perfusion may inhibit collagen deposition in the stomas and thus reduce the tensile strength. Compact collagen deposition developed in the anastomosis of groups A1, A2 and B, with the former group displaying improved bud organization and repair of mucous membranes. Following the rise of the perfusion temperature, the ability of collagenous fiber formation was weakened by the reduced and irregular collagen deposition. Furthermore, inflammatory cell infiltration was observed in the stomas of group D. Together, these results demonstrated the inhibition of both the formation of collagenous fiber and anastomotic healing.

Although perfusion using the open technique is believed to provide optimal thermal homogeneity and homogeneous distribution of chemotherapy in a swine model of IEPCHIP, we performed a closed technique intraoperatively for the following reasons. First, our machine was a closed system, which is suitable for the maintenance of a stable temperature, and the perfusion temperature may decrease at a greater rate in an open environment perfusion. Also, a closed HIPEC was used to reduce the difference in the temperature between the inflow and outflow. Second, the closed technique achieved higher temperatures within the diaphragmatic area, while the open technique has been shown to result in higher temperatures in the mid and lower abdomen [35]. Most gastric cancers reside between the diaphragmatic area and the middle abdomen, where higher temperatures are achieved with the closed technique. Third, we gently massaged the abdominal wall during the perfusion, which made the distribution of the perfusion fluid and heat conduction equal. Fourth, operator exposure to chemotherapeutic agents and environmental pollution must be considered with the open technique [36]. Fifth, the time of general anesthesia is extended for the open technique, which may increase the risk of the associated surgery.

The efficacy and safety of intraperitoneal hyperthermic perfusion chemotherapy are influenced by the following factors [37]: the dose and concentration of the chemotherapy used; the intraperitoneal perfusion temperature and duration; the perfusion times and the osmotic pressure of the treatment fluid. Currently, no large-scale multicenter randomized controlled trials examining different chemotherapy schemes and different combinations of hyperthermia and chemotherapy have been completed to determine the best chemotherapy scheme for peritoneal carcinomatosis of gastric cancer, specifically in Asian populations. The design of such a study is very complex, as it must fully simulate the combined effect of different temperatures and different commonly used chemotherapeutic agents. However, after evaluating appropriate perfusion temperatures and the optimal scheme of chemotherapy, we should be able to construct a better model of perfusion and fully evaluate the clinical effects. This approach would also save animals and reduce their suffering. Initially, it is important to focus on the appropriate perfusion temperature in the absence of chemotherapy, as suitable temperatures are the basis of these studies and their clinical transformation. Furthermore, efficacy studies should be performed with acceptable complications using a certain temperature; for example, because a perfusion temperature higher than 42.5 ± 0.5°C with or without chemotherapy was able to kill free cancer cells, the safety of perfusion at different temperatures should be considered.

As the survival rates have improved after cytoreductive surgery plus IEPCHIP for gastric cancer, the toxicity of additional chemotherapy should be monitored closely. In a meta-analysis of randomized controlled trials on hyperthermic intraperitoneal chemotherapy for resectable gastric cancer, the postoperative mortality rates of 6 trials were 2.6% (10/385) [11]. In a multi-institutional study of 1,290 patients that examined the treatment of peritoneal carcinomatosis of non-ovarian origin by cytoreductive surgery and perioperative intraperitoneal chemotherapy, the mortality rate was 4.1% [28]. In another multi-institutional study examining cytoreductive surgery combined with intraoperative and postoperative intraperitoneal chemotherapy for peritoneal carcinomatosis from colorectal cancer, the related mortality rate was reported to be 4% (20/506) [29].

Additional chemotherapy may be excessively risky for these patients, and the possible increase in cancer stem cell number after chemotherapy is of further concern [38]. Hyperthermic perfusion can also be effective, allowing a better quality of life without some of the complications observed after procedures for advanced gastric cancer. Besides, cytoreductive surgery with hyperthermic intraperitoneal chemotherapy prolongs survival at considerable morbidity and mortality in elderly patients [39]. Hyperthermic intraperitoneal perfusion without chemotherapy may be especially suitable for elderly patients with a poor performance status or those who cannot tolerate chemotherapy. Therefore, we performed intraoperative and early postoperative continuous hyperthermic intraperitoneal perfusion without chemotherapy on 34 patients with advanced gastric cancer since 2011.

For the above-mentioned reasons, the current study focused on determining the acceptable temperature range, which is the basis of hyperthermic intraperitoneal perfusion with or without chemotherapy.

## Conclusions

We found that IEPCHIP at 42.5 ± 0.5°C did not significantly harm the recipient pigs in terms of their vital signs, liver function, renal function and anastomotic healing. Furthermore, there was no added risk of anastomotic leakage and abdominal adhesion. Thus, perfusion at 42.5 ± 0.5°C was safe and appropriate for IEPCHIP, whereas perfusion at 43.5 ± 0.5°C or 44.5 ± 0.5°C was harmful and shown to significantly worsen anastomotic healing.

## Abbreviations

IEPCHIP: Intraoperative and early postoperative continuous hyperthermic intraperitoneal perfusion; ALT: Glutamic-pyruvic transaminase; TBIL: Total bilirubin; ALB: albumin; CR: Creatinine; BUN: Blood urea nitrogen.

## Competing interests

The authors declare no competing interests.

## Authors’ contributions

SL and YZ contributed equally to this study. YH and PW are both corresponding authors and they conceived, designed and partially conducted the study; these authors also played a major role in the revision of the manuscript. SL participated in the design of the study and performed the statistical analyses; SL and YZ helped to conduct the study and draft the manuscript; JS provided technical support and was involved in the drafting of the manuscript. All authors read and approved the final manuscript.

## References

[B1] Jemal A Bray F Center MM Ferlay J Ward E Forman D Global cancer statistics CA Cancer J Clin 2011 61 69 90 10.3322/caac.20107 21296855

[B2] Al-Shammaa HA Li Y Yonemura Y Current status and future strategies of cytoreductive surgery plus intraperitoneal hyperthermic chemotherapy for peritoneal carcinomatosis World J Gastroenterol 2008 14 1159 1166 10.3748/wjg.14.1159 18300340 PMC2690662

[B3] Roviello F Caruso S Marrelli D Pedrazzani C Neri A De Stefano A Pinto E Treatment of peritoneal carcinomatosis with cytoreductive surgery and hyperthermic intraperitoneal chemotherapy: state of the art and future developments Surg Oncol 2011 20 e38 e54 10.1016/j.suronc.2010.09.002 20888755

[B4] Yonemura Y Kawamura T Bandou E Tsukiyama G Endou Y Miura M The natural history of free cancer cells in the peritoneal cavity Recent Results Cancer Res 2007 169 11 23 17506246 10.1007/978-3-540-30760-0_2

[B5] Yang XJ Huang CQ Suo T Mei LJ Yang GL Cheng FL Zhou YF Xiong B Yonemura Y Li Y Cytoreductive surgery and hyperthermic intraperitoneal chemotherapy improves survival of patients with peritoneal carcinomatosis from gastric cancer: final results of a phase III randomized clinical trial Ann Surg Oncol 2011 18 1575 1581 10.1245/s10434-011-1631-5 21431408 PMC3087875

[B6] Yonemura Y Fujimura T Nishimura G Falla R Sawa T Katayama K Tsugawa K Fushida S Miyazaki I Tanaka M Effects of intraoperative chemohyperthermia in patients with gastric cancer with peritoneal dissemination Surgery 1996 119 437 444 10.1016/S0039-6060(96)80145-0 8644010

[B7] Tang L Mei LJ Yang XJ Huang CQ Zhou YF Yonemura Y Li Y Cytoreductive surgery plus hyperthermic intraperitoneal chemotherapy improves survival of gastric cancer with peritoneal carcinomatosis: evidence from an experimental study J Transl Med 2011 9 53 10.1186/1479-5876-9-53 21548973 PMC3098163

[B8] Glehen O Gilly FN Arvieux C Cotte E Boutitie F Mansvelt B Bereder JM Lorimier G Quenet F Elias D Peritoneal carcinomatosis from gastric cancer: a multi-institutional study of 159 patients treated by cytoreductive surgery combined with perioperative intraperitoneal chemotherapy Ann Surg Oncol 2010 17 2370 2377 10.1245/s10434-010-1039-7 20336386

[B9] Esquivel J Sticca R Sugarbaker P Levine E Yan TD Alexander R Baratti D Bartlett D Barone R Barrios P Cytoreductive surgery and hyperthermic intraperitoneal chemotherapy in the management of peritoneal surface malignancies of colonic origin: a consensus statement. Society of Surgical Oncology Ann Surg Oncol 2007 14 128 133 17072675 10.1245/s10434-006-9185-7

[B10] Klaver YL Hendriks T Lomme RM Rutten HJ Bleichrodt RP De Hingh IH Intraoperative versus early postoperative intraperitoneal chemotherapy after cytoreduction for colorectal peritoneal carcinomatosis: an experimental study Ann Surg Oncol 2012 19 Suppl 3 S475 S482 21837528 10.1245/s10434-011-1984-9

[B11] Yan TD Black D Sugarbaker PH Zhu J Yonemura Y Petrou G Morris DL A systematic review and meta-analysis of the randomized controlled trials on adjuvant intraperitoneal chemotherapy for resectable gastric cancer Ann Surg Oncol 2007 14 2702 2713 10.1245/s10434-007-9487-4 17653801

[B12] Brucher BL Piso P Verwaal V Esquivel J Derraco M Yonemura Y Gonzalez-Moreno S Pelz J Konigsrainer A Strohlein M Peritoneal carcinomatosis: cytoreductive surgery and HIPEC–overview and basics Cancer Invest 2012 30 209 224 10.3109/07357907.2012.654871 22360361

[B13] Uzunkoy A Bolukbas C Horoz M Bolukbas FF Kocyigit A The optimal starting time of postoperative intraperitoneal mitomycin-C therapy with preserved intestinal wound healing BMC Cancer 2005 5 31 10.1186/1471-2407-5-31 15801977 PMC1079801

[B14] Khatri VP Cytoreductive surgery and hyperthermic intraperitoneal chemotherapy for colorectal cancer: a panacea or just an obstacle course for the patient? J Clin Oncol 2010 28 5 7 10.1200/JCO.2009.24.8500 19917856

[B15] Kusamura S Baratti D Deraco M Multidimensional analysis of the learning curve for cytoreductive surgery and hyperthermic intraperitoneal chemotherapy in peritoneal surface malignancies Ann Surg 2012 255 348 356 10.1097/SLA.0b013e3182436c28 22202584

[B16] Kusamura S Younan R Baratti D Costanzo P Favaro M Gavazzi C Deraco M Cytoreductive surgery followed by intraperitoneal hyperthermic perfusion: analysis of morbidity and mortality in 209 peritoneal surface malignancies treated with closed abdomen technique Cancer 2006 106 1144 1153 10.1002/cncr.21708 16456817

[B17] Verwaal VJ Van Tinteren H Ruth SV Zoetmulder FA Toxicity of cytoreductive surgery and hyperthermic intra-peritoneal chemotherapy J Surg Oncol 2004 85 61 67 10.1002/jso.20013 14755505

[B18] Kusamura S Baratti D Antonucci A Younan R Laterza B Oliva GD Gavazzi C Deraco M Incidence of postoperative pancreatic fistula and hyperamylasemia after cytoreductive surgery and hyperthermic intraperitoneal chemotherapy Ann Surg Oncol 2007 14 3443 3452 10.1245/s10434-007-9551-0 17909918

[B19] Tsilimparis N Bockelmann C Raue W Menenakos C Perez S Rau B Hartmann J Quality of life in patients after cytoreductive surgery and hyperthermic intraperitoneal chemotherapy: is it worth the risk? Ann Surg Oncol 2013 20 226 232 10.1245/s10434-012-2579-9 22868919

[B20] El-Malt M Ceelen W Van den Broecke C Cuvelier C Van Belle S De Hemptinne B Pattyn P Influence of neo-adjuvant chemotherapy with 5-fluorouracil on colonic anastomotic healing: experimental study in rats Acta Chir Belg 2003 103 309 314 12914369 10.1080/00015458.2003.11679430

[B21] Smeenk RM Verwaal VJ Zoetmulder FA Toxicity and mortality of cytoreduction and intraoperative hyperthermic intraperitoneal chemotherapy in pseudomyxoma peritonei–a report of 103 procedures Eur J Surg Oncol 2006 32 186 190 10.1016/j.ejso.2005.08.009 16303281

[B22] Van der Speeten K Stuart OA Sugarbaker PH Pharmacokinetics and pharmacodynamics of perioperative cancer chemotherapy in peritoneal surface malignancy Cancer J 2009 15 216 224 10.1097/PPO.0b013e3181a58d95 19556908

[B23] Sugarbaker PH Observations concerning cancer spread within the peritoneal cavity and concepts supporting an ordered pathophysiology Cancer Treat Res 1996 82 79 100 10.1007/978-1-4613-1247-5_6 8849945

[B24] Murray TG Cicciarelli N McCabe CM Ksander B Feuer W Schiffman J Mieler WF O’Brien JM In vitro efficacy of carboplatin and hyperthermia in a murine retinoblastoma cell line Invest Ophthalmol Vis Sci 1997 38 2516 2522 9375570

[B25] Klaver YL Hendriks T Lomme RM Rutten HJ Bleichrodt RP De Hingh IH Hyperthermia and intraperitoneal chemotherapy for the treatment of peritoneal carcinomatosis: an experimental study Ann Surg 2011 254 125 130 10.1097/SLA.0b013e3182197102 21502859

[B26] Deng HJ Wei ZG Zhen L Li GX Uang XC Qing SH Clinical application of perioperative continuous hyperthermic peritoneal perfusion chemotherapy for gastric cancer Nan Fang Yi Ke Da Xue Xue Bao 2009 29 295 297 19246304

[B27] Jacquet P Averbach A Stephens AD Stuart OA Chang D Sugarbaker PH Heated intraoperative intraperitoneal mitomycin C and early postoperative intraperitoneal 5-fluorouracil: pharmacokinetic studies Oncology 1998 55 130 138 10.1159/000011847 9499187

[B28] Glehen O Gilly FN Boutitie F Bereder JM Quenet F Sideris L Mansvelt B Lorimier G Msika S Elias D Toward curative treatment of peritoneal carcinomatosis from nonovarian origin by cytoreductive surgery combined with perioperative intraperitoneal chemotherapy: a multi-institutional study of 1,290 patients Cancer 2010 116 5608 5618 10.1002/cncr.25356 20737573

[B29] Glehen O Kwiatkowski F Sugarbaker PH Elias D Levine EA De Simone M Barone R Yonemura Y Cavaliere F Quenet F Cytoreductive surgery combined with perioperative intraperitoneal chemotherapy for the management of peritoneal carcinomatosis from colorectal cancer: a multi-institutional study J Clin Oncol 2004 22 3284 3292 10.1200/JCO.2004.10.012 15310771

[B30] Bouquet W Ceelen W Adriaens E Almeida A Quinten T De Vos F Pattyn P Peeters M Remon JP Vervaet C In vivo toxicity and bioavailability of Taxol and a paclitaxel/beta-cyclodextrin formulation in a rat model during HIPEC Ann Surg Oncol 2010 17 2510 2517 10.1245/s10434-010-1028-x 20339948

[B31] Das A Heat stress-induced hepatotoxicity and its prevention by resveratrol in rats Toxicol Mech Methods 2011 21 393 399 10.3109/15376516.2010.550016 21426263

[B32] Kehoe SM Williams NL Yakubu R Levine DA Chi DS Sabbatini PJ Aghajanian CA Barakat RR Abu-Rustum NR Incidence of intestinal obstruction following intraperitoneal chemotherapy for ovarian tubal and peritoneal malignancies Gynecol Oncol 2009 113 228 232 10.1016/j.ygyno.2009.01.016 19254808

[B33] Yamaguchi R Terashima H Yoneyama S Tadano S Ohkohchi N Effects of platelet-rich plasma on intestinal anastomotic healing in rats: PRP concentration is a key factor J Surg Res 2012 173 258 266 10.1016/j.jss.2010.10.001 21074782

[B34] Aarts F Bleichrodt RP De Man B Lomme R Boerman OC Hendriks T The effects of adjuvant experimental radioimmunotherapy and hyperthermic intraperitoneal chemotherapy on intestinal and abdominal healing after cytoreductive surgery for peritoneal carcinomatosis in the rat Ann Surg Oncol 2008 15 3299 3307 10.1245/s10434-008-0070-4 18712445

[B35] Ortega-Deballon P Facy O Jambet S Magnin G Cotte E Beltramo JL Chauffert B Rat P Which method to deliver hyperthermic intraperitoneal chemotherapy with oxaliplatin? An experimental comparison of open and closed techniques Ann Surg Oncol 2010 17 1957 1963 10.1245/s10434-010-0937-z 20143265

[B36] Simon L Halilou MC Gladieff L Gadiou M Herin F Hennebelle I Chatelut E Ferron G Hyperthermic intraoperative intraperitoneal chemotherapy (HIPEC): evaluation, prevention and policies to avoid occupational exposure for operating room personnel Bull Cancer 2009 96 971 977 19762323 10.1684/bdc.2009.0927

[B37] Kusamura S Dominique E Baratti D Younan R Deraco M Drugs, carrier solutions and temperature in hyperthermic intraperitoneal chemotherapy J Surg Oncol 2008 98 247 252 10.1002/jso.21051 18726886

[B38] Dylla SJ Beviglia L Park IK Chartier C Raval J Ngan L Pickell K Aguilar J Lazetic S Smith-Berdan S Colorectal cancer stem cells are enriched in xenogeneic tumors following chemotherapy PLoS One 2008 3 e2428 10.1371/journal.pone.0002428 18560594 PMC2413402

[B39] Votanopoulos KI Newman NA Russell G Ihemelandu C Shen P Stewart JH Levine EA Outcomes of Cytoreductive Surgery (CRS) with Hyperthermic Intraperitoneal Chemotherapy (HIPEC) in Patients Older Than 70 Years; Survival Benefit at Considerable Morbidity and Mortality Ann Surg Oncol 2013 10.1245/s10434-013-3053-z PMC3881978 23780382

